# Overcoming the novelty effect on YouTube: visibility patterns in evidence-based psychotherapy videos—Findings of a long-term observational study in French-speaking countries

**DOI:** 10.3389/fpsyg.2026.1744748

**Published:** 2026-03-05

**Authors:** Amaury Durpoix, Amaury C. Mengin, Laurence Lalanne, Mario Speranza, Nader Perroud, Marie Riebel, Christophe Moog, Satchel Cohen, Lionel Cailhol, Martin Blay, Sébastien Weibel

**Affiliations:** 1 Psychiatry, Mental Health and Addictology Department, University Hospitals of Strasbourg, Strasbourg, France; 2 Inserm UMR_S 1329, Team Addictions, Centre de recherche en biomédecine de Strasbourg, Strasbourg, France; 3 Faculty of Medicine, Strasbourg University, Strasbourg, France; 4 Inserm UMR_S 1329, Equipe Psychiatrie, Centre de recherche en biomédecine de Strasbourg, Strasbourg, France; 5 Psychiatry of Child and Adolescent, Hospital Center of Versailles, Le Chesnay, France; 6 Paris-Saclay University, UVSQ, Inserm, CESP UMR1018, Team DevPsy, Versailles, France; 7 Department of Psychiatry, Geneva University Hospitals, Geneva, Switzerland; 8 Faculty of Medicine, University of Geneva, Geneva, Switzerland; 9 Faculty of Psychology, Strasbourg University, Strasbourg, France; 10 Department of Psychiatry and Research Center, Institut Universitaire de Santé Mentale de Montréal, Montreal, QC, Canada; 11 Department of Psychiatry and Addiction, Université de Montréal, Montreal, QC, Canada; 12 ADDIPSY, Santé Basque Développement group, Lyon, France; 13 Claude Bernard Lyon 1 University, Lyon, France

**Keywords:** compassion-focused therapy, dialectical behavior therapy, digital tools, e-psychiatry, France and French-speaking countries, novelty effect, YouTube videos

## Abstract

**Introduction:**

As the second most visited website globally, YouTube offers a major opportunity for disseminating mental health knowledge and reducing stigma around psychotherapy. However, visibility remains a key challenge, particularly in the long-term because YouTube algorithm favors novelty over relevance. This novelty effect may discourage scientifically trained psychotherapists from publishing high-quality content due to its limited lifespan. Yet, videos created by clinicians can be directly recommended during in-person therapy or professional exchanges, potentially bypassing algorithmic limitations and maintaining long-term visibility. This study investigated whether such videos maintain long-term viewership and explored the factors influencing their engagement.

**Methods:**

We analyzed viewership data automatically collected by YouTube for 51 videos posted between 2018 and 2022 on the GREMO HUS channel. All videos were developed by academic psychotherapists, grounded in evidence-based practices and organized into three playlists: “Meditation & Relaxation,” “DBT Skills,” “Compassion Focused Therapy.”

**Results:**

Average monthly channel views increased significantly from 365.3 in 2019–2020 to 1,669.5 in 2021–2022 (*p* < 0.001), alongside a rise in average viewer retention from 24.7 to 33.9% (*p* < 0.001). At the video level, monthly views increased both with time since publication (*p* < 0.001) and by calendar year (from 3.8 views per month in 2019 to 45.9 per month in 2022; *p* < 0.001). Video length was positively associated with views for meditative videos, but negatively for “DBT skills” videos. Viewer retention improved with the inclusion of role-play scenes but not by speech rate, except for “Meditation and Relaxation.” User comments frequently mentioned the videos' utility in face-to-face psychotherapy and professional training.

**Discussion:**

Our findings demonstrate that evidence-based videos can overcome the novelty effect commonly associated with YouTube when embedded in real-life therapeutic practices. This suggests a hybrid dissemination model—leveraging both digital platforms and offline clinical networks—may effectively bypass algorithmic limitations. Future research should investigate the generalizability of these findings to other healthcare domains and platforms, and further explore the mechanisms through which professional recommendation influences digital engagement.

## Highlights

- Novelty effect of YouTube can be overcome by psychotherapy videos
- Real-life recommendations can bypass algorithmic limitations of YouTube
- Health professionals can easily realize real-life recommendations

## Introduction

As the second most visited website worldwide after Google (Gaudiaut, 2023), YouTube offers a major opportunity for the dissemination of medical knowledge (Drozd et al., 2018). Since the rise of the internet, most individuals have turned to online sources for health-related information (Diaz et al., 2002). Among various digital tools, YouTube stands out due to its anonymity and free access-features that are particularly valuable in psychiatry, where stigma and limited public understanding remain persistent challenges (Parcesepe and Cabassa, 2013). Stigmatization of mental health, sometimes even originating from healthcare professionals themselves (Henderson et al., 2014; Vistorte et al., 2018), is a significant barrier that deters individuals from seeking care out of fear of judgment (Jaeschke et al., 2021; Kohn et al., 2004; Sareen et al., 2007). Notably, 85% of young patients identify YouTube as a key platform for mental health information (Lal et al., 2015), and several studies have emphasized its relevance in promoting mental health literacy (MacLean et al., 2017; Woo et al., 2018).

Despite this potential, visibility remains a major challenge when publishing videos on YouTube. The vast majority of content receives very few views (Cha et al., 2007), and videos that do gain attention tend to peak rapidly due to the “novelty effect.” On average, most views are accumulated within the first 3 days of publication (Figueiredo et al., 2011). This novelty effect, common across digital tools (Shin et al., 2019), is reinforced by YouTube's algorithms, which tend to promote content primarily within the first 30 days of publication (Zhou et al., 2016). Thus, recency becomes a key determinant of visibility, often outweighing long-term relevance or accuracy. Given that producing a single video can take up to 12 h (Maynard, 2021), this limited exposure can be particularly discouraging for professionals who balance clinical or academic responsibilities.

This algorithmic bias has important consequences for the quality of information available online. Previous studies have highlighted the highly variable quality and reliability of medical information available on YouTube, emphasizing the need for systematic evaluation of health-related video content across clinical domains (Tezcan and Akyildiz Tezcan, 2024). While academics and healthcare professionals produce scientifically sound content, most YouTube videos are created by individuals without formal scientific training (Dagar and Falcone, 2020). Their more frequent posting schedules result in newer videos being algorithmically favored, despite a higher risk of misinformation. In mental health, this risk is particularly acute, as some individuals share personal experiences without sufficient clinical insight—for example, in the context of anorexia (Syed-Abdul et al., 2013). Furthermore, even professionals often lack adequate training in evidence-based psychotherapies. A gap between scientific knowledge and its clinical application has been observed in psychotherapy for various disorders, including addiction (Carroll, 2014), PTSD (Pittig et al., 2019), and borderline personality disorder (Blay et al., 2023; Iliakis et al., 2019). In several countries, including France, evidence-based psychotherapies continue to be debated (Amouroux et al., 2022; Amouroux and Zaslawski, 2019; Durpoix, 2022), often due to insufficient integration between research and practice (Lilienfeld et al., 2013, 2015).

In this context, YouTube has emerged as both a dissemination tool and a battleground. While the platform can enhance access to scientifically validated interventions, its algorithm tends to favor novelty and popularity over reliability and long-term relevance (Halim et al., 2022; Zhou et al., 2016). Overcoming this limitation could significantly enhance the reach of evidence-based psychotherapies. Similar to self-help books (Cuijpers, 1997; Scholtens, 2024), video-based psychotherapies have shown clinical efficacy in randomized controlled trials (Ciuca et al., 2018; Mengin et al., 2024) and meta-analysis (Klimczak et al., 2023; van Leeuwen et al., 2021). YouTube could thus serve as a platform to improve access to such therapies. In psychoeducation, a randomized controlled trial showed that YouTube videos can reduce depressive symptoms (Otu et al., 2023). During the COVID-19 lockdown, initiatives such as the GREMO HUS YouTube channel, led by academic psychotherapists, proved helpful to patients (Durpoix et al., 2021).

Despite this potential, little is known about the long-term visibility of educational health content on YouTube. Existing studies in psychiatry have focused on average viewership across entire channels (Lam et al., 2017; Michalak et al., 2017), without exploring how individual videos perform over time. One hypothesis worth testing is that real-life recommendations during psychotherapy may provide an alternative pathway to sustained visibility, bypassing algorithmic limitations. Accordingly, the primary objective of this study was to analyze the viewership trends of “GREMO HUS” YouTube videos over a 4-year period. The secondary objective was to explore factors associated with this viewership.

## Methods

### Viewers

The data included in this observational study concerned every viewer who watched any of the GREMO HUS videos between the channel's launch in June 2018 and December 2022. The data is collected automatically and anonymously by YouTube. Any Internet user agrees to the collection and analysis of data for the purpose of improving the user experience.

Viewers were mainly from France (90.7%), women (64.4%) and aged 25–34 (23.8%). The other countries of viewers were French-speaking with Canada (5.97%), Morocco (0.85%), Algeria (0.68%), Switzerland (0.51%), Belgium (0.34%), Tunisia (0.17%), Senegal (0.17%), Luxembourg (0.09%), except Germany (0.34%), Brazil (0.09%), and Iran (0.09%). The other age groups were 18–24 (9.8%), 35–44 (23.5%), 45–54 (22.1%), 55–64 (11.2), and >65 (9.6%). The proportion of the age group < 18 was not provided by YouTube.

### Procedure

This observational study analyzed the data obtained from YouTube in the “Analytics Data” tab of the “GREMO HUS” channel account. YouTube automatically provides these data, which include general socio-demographic characteristics of viewers (average age, sex ratio, and geographic origin), viewing characteristics (number of views, duration of viewing, number of likes or dislikes, number of shares and impressions) and video characteristics (duration, type, and speech rate).

This study was approved by the ethics committee of the Strasbourg's Medicine Faculty (CE-2023-121).

### Therapy model

The “GREMO HUS” channel conveys content from two evidence-based therapies (Millard et al., 2023; Storebø et al., 2020), which include a group modality:

- Dialectical Behavior Therapy (DBT) belongs to the 3rd wave of Cognitive-Behavioral Therapies and represents the therapy with the highest evidence level of efficacy in BPD (Storebø et al., 2020). It aims to reduce emotional dysregulation by balancing the acceptance skills, taught in the mindfulness and distress tolerance modules, with the change skills taught in the emotional regulation and interpersonal effectiveness modules (Linehan, 1993). DBT has been practiced and studied in Strasbourg since 2018 (Bemmouna et al., 2021; Durpoix et al., 2023).
- Compassion-Focused Therapy (CFT) involves working more specifically on compassion skills, both toward others and toward oneself (Gilbert, 2010). Aimed at reducing self-criticism, CFT is usually practiced transdiagnostically. It has been practiced in Strasbourg since 2020 and has also been the subject of several studies (Riebel et al., 2023; Riebel and Weiner, 2023).

### Videos

To improve knowledge transfer in psychotherapy, the YouTube channel “GREMO HUS” was created in June 2018 (“GREMO HUS” for Groupe de Régulation Emotionnelle des Hôpitaux Universitaires de Strasbourg) in a French hospital-university setting in June 2018: https://www.youtube.com/@gremohus8612. The videos were produced by academic therapists practicing DBT and CFT in the psychiatry department of the University Hospital of Strasbourg. Each video was recorded by the professionals depending on their availability, without funding. They are entirely in French and uploaded separately on the YouTube channel. They are not paid by YouTube. A significant number of videos were produced during the first wave of COVID-19. Posted mainly during the confinement to address the interruption of group sessions, the videos were primarily intended to support patients' skills learning in therapy. Additionally, they served to promote these therapies to patients, professionals, and the public. They were recommended during training sessions, congresses, association meetings (e.g., French-speaking association for DBT, Family Connections) and media interviews.

Fifty-one videos are open access. They last between 2 min 10 s and 27 min 26 s (mean = 13 min, standard deviation = 5 min). They are grouped into three different playlists (see Table 1 for global characteristics and Supplementary material for individual characteristics). The “Meditation and Relaxation” and “Compassion-Focused Therapy” playlists are meditative and include mindfulness, relaxation, or compassion imagery tasks. The “DBT Skills” playlist is informative, explaining DBT skills in theory and sometimes in practice through role-playing scenes that add a ludic side. In this playlist, a summary is available below the video so that viewers can choose to watch only certain parts of the video.

**Table 1:** Global characteristics of open-access videos.

**Date of publication**	**Title of videos**
**Playlist “Meditation and relaxation” (*****N*** = **17, M** = **10 min 52)**	
2018	Bodyscan, Brief meditation 2min, Body Scan, Sleep preparation, Meditation 5 min Meditation to calm the mind, Mindful walking, Relaxation 6 min, Relaxation 2 (6 min)
2019	Meditation to calm the mind, Mindful walking, Relaxation 6 min, Relaxation 2 (6 min) Relaxation with music (20 min)
2020	Thought meditation−10 min, Introduction to mindfulness and relaxation practices, Mindful Walk−8 min, Muscle relaxation−15 min, Mindfulness—Body scan−22 min
2021	Relaxation through Schultz's autogenous training, Body Scan—Meditation 20 min
**Playlist “DBT Skills” (*****N*** = **20, M** = **15 min 53)**	
03-05/2020	When to use DBT skills, Understanding emotions^*^, Fact-Checking^*^, Distraction ACCEPTS, STOP and TIP, Acting the opposite^*^, Problem Solving^*^, Resilience—ABC PLEASE^*^, Priorities & DEAR MAN^*^, Radical acceptance^*^, Commitment vs. Obstinacy^*^, IMPROVE, GIVE & FAST^*^, Validation^*^
Others	Mindfulness 1/2^*^ (09/2020), Mindfulness 2/2^*^ (09/2020), Building relationships (12/2020), Forgiveness (08/2021), Open Mind (02/22), How to complete a daily self-observation form (11/22)
**Playlist “Compassion-Focused therapy” (*****N*** = **6, M** = **10 min 30)**	
02/2021	Soothing breathing rhythm, Soothing breathing
03/2021	Place of Serenity (short version), Color of Compassion, Ideal of compassion
04/2021	Compassionate self

^*^Presence of role-playing scenes.

In addition, 25 videos are private access, as they are intended exclusively for professionals. Two were loaded in 2020; 22 in 2021; two in 2022. The data didn't include these videos, except to see the proportion of views represented by open access videos.

### Statistical analysis

Data were analyzed using IBM SPSS Statistics version 27. We performed a descriptive analysis of users' socio-demographic data, video characteristics and viewing. Prior to inferential analyses, the normality of data distributions was assessed using Shapiro–Wilk tests, histograms, and Q–Q plots. As most of the key variables (e.g., monthly views, retention times) deviated significantly from normality (*p* < 0.05) and included outliers or skewed distributions, non-parametric tests were selected. These tests are more robust to violations of normality and are appropriate for small to moderate sample sizes or when data do not meet assumptions of parametric tests (Field, 2024; Ghasemi and Zahediasl, 2012). Statistical significance was considered for any *p*-value below 0.05.

To analyze viewing over time, non-parametric tests were applied first at the channel level, then at the individual videos level. For the channel, monthly views and retention were compared between 2019–2020 and 2021–2022 using Wilcoxon tests. For the videos, we assessed the relationship between monthly views and video age (in months since publication) using Spearman correlation, and compared views across calendar years using Friedman test as ANOVA alternative. When significant differences emerged, *post-hoc* tests were conducted using Wilcoxon tests with Bonferroni correction. These tests were conducted for the full set of videos and separately for the three video playlists.

To explore the influence of video characteristics on the viewing, monthly views of videos were compared by the “pre-click” characteristic visible before clicking on video, namely video length (Kruskal–Wallis test). Then, the relation between total video views and retention percentage of videos in 2022 was examined (Spearman correlation). Finally, the retention percentages of videos in 2022 were compared by the “post-click” characteristics visible after the click, namely speech rate (Kruskal–Wallis test) and the presence/absence of a role-playing scene (Mann–Whitney test).

To identify indicators of real-life influence—such as mentions of therapeutic contexts or external recommendations—a qualitative analysis of comments was conducted independently by two raters. Each coded the comments separately, then discrepancies were discussed to reach consensus and finalize the categorization scheme.

## Results

### Descriptive results on viewing

Since its creation in June 2018, the GREMO HUS channel has accumulated 48,735 views, 47,556 for open-access videos (97.6%), with an average of 1,112 views/video (SD = 1,195) and a maximum of 5,931 views for one video (“Understanding Emotion”). The open-access videos received an average of 34.5 views per month (SD = 44.95): 13.0 views per month for the “Meditation and Relaxation” videos (SD = 16.3), 63.1 for the “Dialectical and Behavioral Therapy Skills” videos (SD = 55.1), and 21.3 for the “Compassion-Focused Therapy” videos (SD = 17.7). Average retention per view was 32.4% of the video (maximum = 47.36% for “Sleep preparation”) and 4 min 47 (maximum = 8 min 09 for “Body Scan—Meditation 20 min”). Full retention was 24.1% of views on average (maximum = 35.8% for “Mindfulness 1/2”). The channel accumulated 3,903.3 h of viewing, 883 likes, 17 dislikes, 1,413 video shares and 1,277 followers.

Views were mainly performed on mobile (50.3%), followed by computer (42.7%), tablet (4.5%), and television (2.4%). They were attracted by diverse sources. The majority were linked to research on internet (“external links” = 19.3%) or on YouTube through different pages (“YouTube searches” = 16.2%, “channel pages” = 12.4%, “playlist” = 15.2%, “playlist pages” = 7%). The others were linked to suggestions from YouTube algorithms (“video suggestion” = 13%, “notifications” = 0.3%), from previously viewed videos (“end screens” = 1.2%, “videos cards and annotations” = 0.1%), or from other sources (“direct or unknown sources” = 7.2%, “navigation features” = 6.2%, “other YouTube features” = 1.9%).

### Analysis of the viewing evolution

**Figure 1:**
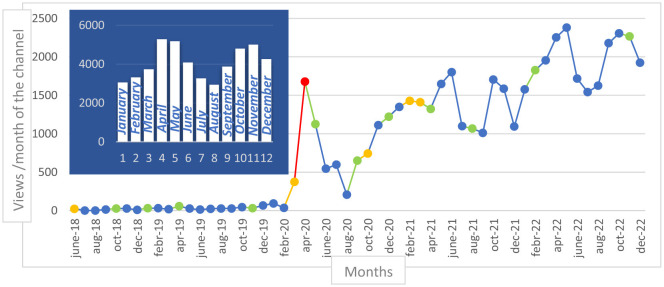
Evolution of views on the channel across the June 2018-December 2022 period and per month. blue = 0 new video, green = 1–2 news videos, orange = 3–4 news videos, red > 5 news videos.

Concerning the channel, views gradually increased over time with a peak during the lockdown in April 2020 (Figure 1). Views were highest in the April–May and October-November periods and lowest in the January-February and July–August periods. Compared to the 2019–2020 period, the channel recorded an increased activity in 2021–2022 for most criteria (Table 2). This increase mainly concerned the French-speaking countries. In Canada, the number of views increased particularly after a conference in June 2021 where we communicated on the GREMO HUS YouTube channel. This country had the greatest improvement of views ( × 36) in 2021–2022 compared to 2019–2020.

**Table 2:** Comparison of monthly average viewing of the channel between 2019–2020 and 2021–2022.

**Name of data**	**2019–2020**	**2021–2022**	**Evolution**
Mean views/month	365.3 (SD = 504.4)	1,669.5 (SD = 476.2)	+357%^***^
Mean retention percentage/view	24.7% (SD = 6.1)	33.9% (SD = 4.1)	+37%^***^
Mean retention minutes/view	2 min 48 (SD = 1 min 05)	5 min 03 (SD = 0 min 35)	+80%^***^
Mean viewing time/month	22.2 h (SD = 30.2)	140.3 h (SD = 37.9)	+532%^***^

^***^p < 0.001.

SD, standard deviation.

**Figure 2:**
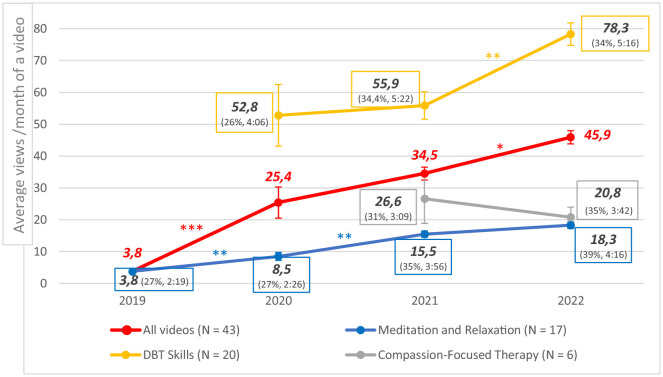
Average monthly views for a video (+ average retention of views in percentage and in minutes). ***p < 0.001 ; **p < 0.01 ; *p < 0.05.

Concerning the monthly video views by calendar year (Figure 2), the Friedman test showed a statistically significant increase with years (χ^2^ = 27.4, *p* < 0.001), between 2019 (mean rank = 1.08), 2020 (mean rank = 2.25), 2021 (mean rank = 2.92) and 2022 (mean rank = 3.75). The number of monthly views increased over the years for “Meditation and Relaxation” (χ^2^ = 27.6, *p* < 0.001) and “DBT Skills” videos (χ^2^ = 7.4, *p* = 0.03), but remained stable for “Compassion-Focused Therapy” videos (χ^2^ = 0.818, *p* = 0.366). *Post-hoc* tests showed statistically significant increases between 2019 and 2020 (*p* < 0.001) and between 2021 and 2022 (*p* = 0.006), particularly for “DBT Skills” videos between 2021 and 2022 (*p* = 0.003) and for “Meditation and Relaxation” videos between 2019–2020 (*p* = 0.005) and 2020–2021 (*p* = 0.008).

**Figure 3:**
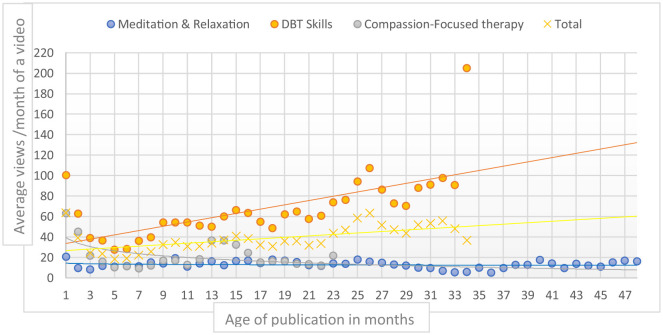
Evolution of video's monthly views over age of publication in months.

Video age (in months since publication) was significantly correlated with monthly views (Spearman's *r* = 0.626, *p* < 0.001), suggesting that older videos tended to receive more views (Figure 3). This correlation was significant for “DBT Skills” videos (*r* = 0.728, *p* < 0.001), but not for “Meditation and Relaxation” videos (*r* = −0.174, *p* = 0.32) nor for “Compassion-Focused Therapy” videos (*r* = −0.079, *p* = 0.73).

### Analysis of influence of video characteristics on viewing

**Figure 4:**
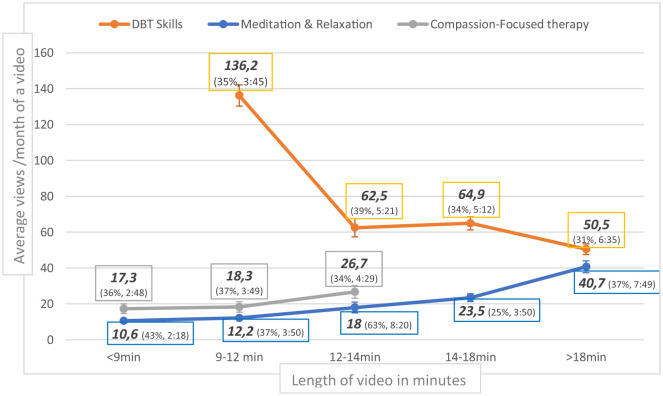
Monthly views of a video by length (in 2022; + average retention of a view in percentage and in minutes).

According to the Kruskal–Wallis test, the number of views was higher for longer videos of “Meditation and Relaxation” (*H* = 38.575, *p* < 0.001) and “Compassion-Focused Therapy” (*H* = 7.144, *p* = 0.03), but lower for “DBT Skills” (*H* = 40.452, *p* < 0.001; Figure 4).

No significant correlation was found between total video views and retention percentage of video (*r* = 0.46, *p* = 0.30—Spearman correlation). When looking at playlist's videos separately, there was a significant positive correlation for “DBT Skills” videos (*r* = 0.332, *p* < 0.001) and a significant negative correlation for “Compassion-Focused Therapy” videos (*r* = −0.327, *p* = 0.005).

**Figure 5:**
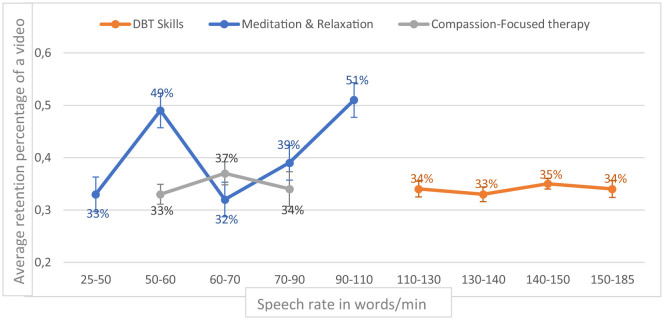
Retention percentage of video by speech rate (words/min; year 2022).

The retention percentage of video significantly increased with speech rate for “Meditation and Relaxation” videos (*H* = 33.537, *p* = < 0.001) according to the Kruskal–Wallis test, but not for “DBT Skills” videos (*H* = 3.861, *p* = 0.28) or “Compassion-Focused Therapy videos” (*H* = 1.569, *p* = 0.46; Figure 5).

The retention percentage of DBT skills videos was higher with role-playing scenes than without (36.9 vs. 29.6%, *p* < 0.001—Mann Whitney test).

### Qualitative analysis of real-life influence on viewing

Fifty-three comments were posted by viewers (74% of thanks, 14% of single question, 12% of advice/criticism). Among them, seven mentioned the help given for their therapy, two mentioned the help given for their professional training, one asked how to find this type of therapy and another how to train as a professional. Three comments from viewers received at least three likes, which are included below for illustration.

- “The tone is calm, the explanations clear, and the vocabulary is well-adapted to the uninitiated. It is a pity that there are no bibliographies to help those who wish to deepen the subject. Care should be taken to provide bibliographies adapted to a range of audiences, from the neophyte to those with in-depth knowledge of the subject.” (7 likes)
- “Thank you, it helps me a lot to organize what I learn in therapy :) few resources in French on the subject, so bravo!!” (5 likes)
- “Great, it really helps me a lot to know how to better use and choose my tools seen in therapy! It's very clear, bravo, don't hesitate to continue, I'm sure it helps a lot of other people!” (3 likes)

## Discussion

### Principal results and comparisons with prior work

This study investigated whether YouTube videos created by academic psychotherapists could maintain long-term visibility for the dissemination of scientific knowledge, despite the novelty effect associated with digital platform. Our findings suggest that such videos—when recommended in real-life clinical or professional contexts—can indeed sustain and even increase their viewership over time. Monthly views on the GREMO HUS channel, as well as on individual videos, rose steadily across the 4-year study period. Notably, this upward trend persisted even during periods of minimal content production, such as in 2022, when only two new videos were uploaded. This pattern suggests that visibility was not solely driven by algorithmic promotion of new content, but was likely supported by offline dissemination strategies.

The “DBT Skills” videos, in particular, showed a marked increase in monthly views, which may reflect their frequent recommendation in psychotherapy or professional training settings. This observation supports our hypothesis that real-life sharing and word-of-mouth dissemination may serve as effective mechanisms for sustaining engagement over time. These findings may encourage scientifically trained clinicians to consider YouTube as a long-term tool for disseminating psychotherapeutic knowledge, especially when combined with complementary real-word communication.

To date, few studies have examined the long-term visibility of mental health-related videos on YouTube, and most have focused on psychoeducational rather than psychotherapeutic content. For instance, a study on psychosis reported that three psychoeducational videos accumulated 4,935 views over 1 year (Lam et al., 2017), while another study on bipolar disorder reported an average monthly views from 324 to 819 over 5 years for a psychoeducation-focused channel (Michalak et al., 2017). These figures are broadly comparable to those observed in our study, where average monthly views increased from 365.3 in 2019–2020 to 1,669.5 in 2021–2022. However, unlike previous research, our study examined the temporal evolution of individual video viewership, providing a more granular understanding of how engagement develops over time. Further research is needed to determine whether these findings can be generalized to other formats and platforms, and to assess whether healthcare professional-created content is more likely to overcome the novelty effect.

A novel contribution of our study is the observation that viewer retention also improved over time. Both the average percentage of video watched and the total viewing duration increased across the study period. This trend may reflect growing awareness of the channel among a targeted and motivated audience, such as patients and healthcare professionals. Although retention metrics are rarely reported in mental health YouTube studies, the viewing durations observed in our sample—typically between 3 and 5 min—are consistent with prior findings in psychoeducational contexts. Previous studies on YouTube reported average viewing times of approximatively 5 min or less (Lam et al., 2017; Michalak et al., 2017), while another study in online education found that students watched an average of 4 min of videos longer than twelve minutes (Guo et al., 2014). Together, these findings suggest that engagement with mental health content on YouTube may follow broader patterns observed in digital learning environments.

Our study also provides several practical insights for professionals seeking to optimize their video content for long-term engagement. The characteristics associated with increased viewership varied according to video type. Longer durations were positively associated with views for meditative videos, whereas shorter formats were more effective for “DBT Skills” content. Viewer retention increased when videos included role-play elements—particularly in DBT-related content—or when the speaking rate was higher, notably in meditation and relaxation videos. These results are consistent with prior research showing that dynamic or playful content enhances engagement on YouTube (Duverger and Steffes, 2012), and that shorter, faster-paced videos are more effective in MOOCs and digital education settings (Guo et al., 2014). Interestingly, the effect of video length appeared to reverse for meditative content, where longer videos attracted more views, possibly reflecting user expectations specific to this genre. Further research could clarify this distinction and help inform content design strategies.

For academic psychotherapists considering video creation, our findings underscore the importance of real-life recommendations. YouTube's algorithms attend to promote videos primarily during the first month after publication (Zhou et al., 2016), after which visibility increasingly depends on word-of-mouth dissemination (Chen et al., 2014). Although our study could not directly quantify the causal impact of offline recommendations, several indicators support their role. First, most users accessed videos through active searching, in contrast to prior studies where algorithmic suggestions predominated (Lam et al., 2017). Second, the socio-demographic profiles of our viewers—predominantly women aged 25 to 45—closely matched the clinical population receiving psychotherapy at our center (Durpoix et al., 2023), differing from psychosis-related content where viewers were mainly male (Lam et al., 2017). Third, user comments frequently referred to psychotherapy sessions or professional training contexts, suggesting that videos were shared within these settings. Together, these observations support the notion that patients and professionals—two groups likely to be influenced by offline recommendations—constituted a substantial portion of the audience.

### Strengths and limitations

This study has several strengths. To our knowledge, it is the first to analyze the long-term viewership dynamics of evidence-based psychotherapy videos on YouTube over a 4-year period. Unlike prior studies relying on static or short-term metrics, our longitudinal approach offers a more realistic depiction of how engagement evolves over time. In addition, the study has strong clinical relevance: the videos were created by academic psychotherapists, grounded in validated therapeutic frameworks, and intended to complement in-person psychotherapy. Finally, the focus on a well-defined clinical population enhances the ecological validity of the findings.

Several limitations should be acknowledged. While the study provided preliminary insights into factors associated with viewership (e.g., video length or role-play elements), it does not allow for a precise causal assessment of the impact of real-life recommendations on visibility. Further studies should aim to better isolate the effects of offline dissemination strategies. Moreover, generalizability may be limited by the single-channel design and the French-language content, which may not reflect broader international usage patterns.

## Conclusion

In conclusion, our findings suggest that carefully designed and clinically grounded YouTube videos can overcome the platform's novelty effect when supported by real-life recommendations and targeted disseminations strategies. This hybrid model—combining digital content with in-person communication—offers a promising avenue for the sustainable dissemination of psychotherapeutic tools. Future research should examine whether these mechanisms extend across languages, clinical contexts, and other digital platforms.

## Data Availability

The raw data supporting the conclusions of this article will be made available by the authors, without undue reservation.
