# Supplementary material for: Overcoming the novelty effect on YouTube: visibility patterns in evidence-based psychotherapy videos—Findings of a long-term observational study in French-speaking countries
Source: Front Psychol. 2026 Mar 5;17:1744748. doi: 10.3389/fpsyg.2026.1744748 (PMC12999454; doi:10.3389/fpsyg.2026.1744748)
Supplement: Supplementary file 1 [file Supplementary_file_1.docx]

**Supplementary material**: Individual characteristics of open-access videos

| Publication | Title | | Length – Speech rate | |
| --- | --- | --- | --- | --- |
| **Playlist "Meditation and relaxation"** | | M = 10min52 – 67 words/min | | |
| **1 -** 14/06/2018  **2** - 15/06  **3** - 24/06  **4** - 24/06  **5** - 11/10  **6** - 06/01/2019  **7** -10/01  **8** - 02/04  **9** - 02/04  **10** - 26/11  **11** - 26/03/2020  **12** - 02/04  **13** -16/10  **14** -16/10  **15** -16/10  **16** -12/02/2021  **17**- 15/04 | Bodyscan  Brief meditation 2min  Body Scan  Sleep preparation  Meditation 5 minutes  Meditation to calm the mind  Mindful walking  Relaxation 6 minutes  Relaxation 2 (6 minutes)  Relaxation with music (20 minutes)  Thought meditation – 10 minutes  Introduction to mindfulness and relaxation practices  Mindful Walk – 8 minutes  Muscle relaxation – 15 minutes  Mindfulness – Body scan – 22 minutes  Relaxation through Schultz's autogenous training  Body Scan – Meditation 20 minutes | | 9:55  2:10  11:35  13:20  5:35  9:30  10:10  6:35  6:40  20:50  10:35  5:00  8:31  15:16  22:41  15:23  20:46 | 28 w/min  106 w/min  40 w/min  55 w/min  46 w/min  80 w/min  63 w/min  68 w/min  59 w/min  ? w/min  73 w/min  98 w/min  85 w/min  64 w/min  61 w/min  56 w/min  86 w/min |
| **Playlist "DBT Skills”** | | M = 15min53 – 146 words/min | | |
| **1** - 21/03/2020  **2** - 27/03  **3** - 04/04  **4** - 07/04  **5** - 07/04  **6** - 12/04  **7** - 12/04  **8** - 12/04  **9** - 19/04  **10** - 25/04  **11** - 25/04  **12** - 28/04  **13** - 01/05  **14** - 23/05  **15** - 12/09  **16** - 12/09  **17** - 12/12  **18** - 07/08/2021  **19** - 02/02/2022  **20** - 15/11 | When to use DBT skills  ER: Understanding emotions *  ER: Fact-Checking *  DT: Distraction with ACCEPTS  DT: STOP and TIP  ER: Acting the opposite *  ER: Problem Solving *  ER: Resilience – ABC PLEASE *  EI: Priorities & DEAR MAN *  DT: Radical acceptance *  DT: Commitment vs. Obstinacy *  DT: IMPROVE  IE: GIVE & FAST *  IE: Validation *  MF: Mindfulness 1/2 *  MF: Mindfulness 2/2 *  IE: Building relationships  IE: Forgiveness  MF: Open Mind  How to complete a daily self-observation form | | 10:04  11 :59  14:54  19:56  20:23  13:49  14:07  19:46  16:14  16:26  13:59  24:31  18:14  14:01  10 :16  13 :55  27 :26  11 :13  16 :59  9 :45 | 125 w/min  141 w/min  148 w/min  135 w/min  134 w/min  121 w/min  135 w/min  123 w/min  142 w/min  139 w/min  146 w/min  126 w/min  126 w/min  144 w/min  164 w/min  181 w/min  156 w/min  175 w/min  175 w/min  176 w/min |
| **Playlist "Compassion-Focused therapy"** | | M = 10min30 – 63 words/min | | |
| **1** - 17/02/2021  **2** - 22/02  **3** - 03/03  **4** - 11/03  **5** - 18/03  **6** - 11/04 | Soothing breathing rhythm  Soothing breathing  Place of Serenity (short version)  Color of Compassion  Ideal of compassion / Compassionate friend  Compassionate self | | 13 :49  6 :35  11 :13  8 :46  13 :00  9 :40 | 50 w/min  61 w/min  69 w/min  59 w/min  69 w/min  71 w/min |

*Legend: ER = Emotional regulation, TD = Distress tolerance, IE = Interpersonal effectiveness, MF = Mindfulness, * = presence of role-playing scenes*
